# Contact tracing: Characteristics of COVID-19 cases that provided contacts

**DOI:** 10.1371/journal.pone.0293208

**Published:** 2023-11-02

**Authors:** Vajeera Dorabawila, Doris Maduka, Virgile Barnes, Nirmala Ramesh, Dina Hoefer

**Affiliations:** Bureau of Surveillance and Data Systems, New York State Department of Health, Albany, New York, United States of America; University of Hong Kong, HONG KONG

## Abstract

This cross-sectional study evaluated COVID-19 contact tracing efforts to identify variations in contact tracing outcomes in different population subgroups. Contact tracing was a critical tool to slow the COVID-19 epidemic. A literature gap evaluating contact tracing elicitation exits, particularly on prioritized groups. We analyzed data from COVID-19 cases linking statewide case management, immunization, laboratory testing, and hospitalization databases in New York State (NYS) outside of New York City from February 1 to November 30, 2021. Focus was cases in home-based residential settings (excluding congregate care) and prioritized groups (educational institutions, large households, close quarters, higher-risk persons, hospitalized). The primary outcome was completed interviews that provided a contact. Of the 550,850 cases interviewed during the study period, 316,645 (57.5%) provided at least one contact. Adults aged 18 to 49 years were most likely to provide contacts than those aged 65 years and older (adjusted odds ratio [aOR], 1.42; 95% confidence interval [CI], 1.39–1.45). Compared to unvaccinated cases, boosted individuals (aOR, 1.61; 95% CI, 1.50–1.73) were most likely to provide contacts, followed by persons with only a primary vaccine series (aOR, 1.3; 95%CI, 1.28–1.33) and partially vaccinated (aOR, 1.21; 95%CI, 1.18–1.24). Repeat cases (aOR, 1.07; 95%CI, 1.01–1.14), pregnant persons (aOR, 1.26; 95% CI, 1,19–1.34), those with underlying conditions (aOR 1.22; 95%CI, 1.20–1.23), and those in K-12 settings (aOR 1.55; 95%CI, 1.50–1.61) were more likely to provide contacts. There was no clear association between hospitalized, while zip code level income may (aOR, 1.006; 95%CI, 1.003, 1.009). Persons from larger households were more likely to provide contacts: aOR for two or more persons vs. one person households ranged from 2.49 to 4.7 (95%CI, 2.20–4.78). Our findings indicate success in eliciting contacts from prioritized groups and identify variable contact elicitation outcomes from different population groups. These results may serve as a tool for future contact tracing efforts.

## Introduction

There were 6,529,084 persons diagnosed with corona virus disease 2019 (COVID-19) as of January 30, 2023, with 3,478,946 from New York State areas other than New York City (referred to as NYS) [1]. Contact tracing “is the process of identifying, assessing, and managing” persons exposed to someone infected with the COVID-19 virus [2]. It is a critical public health tool for slowing the spread of an epidemic and was the case early with the COVID-19 epidemic. An essential aspect of contact tracing is its ability to identify and quarantine contacts while isolating infected cases in a timely manner [3–7]. Early in the epidemic, governments implemented and scaled up contact-tracing programs. While evidence points to success, contact elicitation from cases was a challenge. Wide variations in the percentage of cases providing contact existed and ranged from 21 percent to 87 percent [6, 8–11]. Literature identifying groups with success or challenges in contact elicitation, particularly from prioritized groups [12–14], is absent. This study examined NYS contact-tracing efforts and fills this gap.

## Materials and methods

The primary database, for this cross-sectional study, was the New York State Communicable Disease Case Management System (CDCMS), the system of record for COVID-19 case management in NYS. It was linked to three other databases [15, 16]: Electronic Clinical Laboratory System (ECLRS), NYS Immunization Information System (NYSIIS), and Health Electronic Response Data System (HERDS). All reportable (positive and negative) COVID-19 test results (nucleic acid amplification test [NAAT] or antigen) in NYS are reported to ECLRS. NYSIIS contains COVID-19 vaccination data for NYS residents. HERDS is a statewide, daily electronic survey of COVID-19 hospitalizations at inpatient facilities. American Community Survey 5-year estimates was matched using residential zip code to obtain zip code median income [17].

The analysis period was from February 1, 2021 (the first full month when vaccinations were available [18]) to November 30, 2021 (last month contact tracing was fully functional [12, 13]) (S1 Fig in S1 File). This is a cross-sectional study that covers all reported NYS COVID-19 cases (census) during this period with sufficient observations to draw meaningful conclusions. A case is defined as a positive NAAT or antigen result reported to the ECLRS or to the Local Health Department and recorded in CDCMS [14]. Cases without completed interviews (12.3%) were excluded since interviews are integral to contact elicitation (S2 Fig in S1 File). The analysis excludes persons in congregate care and other unspecified settings (10.6%), restricting to persons living in single- or multi-unit (home-based) residential settings, due to potentially different case elicitation mechanisms in these special settings. Furthermore, persons with missing age, race/ethnicity, and gender (4.8%) as well as missing household size (0.8%) were excluded (4.8%). Sensitivity analysis of exclusions were conducted (S1 File). A contact is defined as a person within 6 feet of a potentially infectious case for at least 15 minutes (cumulative over 24-hour period) starting from 2 days before illness onset and for asymptomatic persons 2 days prior to specimen collection date [7, 13, 14]. The primary outcome was whether a confirmed case reported at least one contact.

Variables identifying the contact-tracing priority groups and demographic and health variables were examined. Priority groups from home-based residential settings included hospitalized, persons in large households living in close quarters, persons working in congregate settings including educational institutions, and people living with or providing care to higher risk persons [7, 13]. At the next level of priority were persons 65 years or older, individuals at higher risk of severe disease, pregnant persons, and those with symptoms. Person reporting Hispanic ethnicity were classified as Hispanic regardless of race and the other race categories are non-Hispanic. The zip code-level median household income [17] was utilized to examine the association with community level factors and specifically socio-economic characteristics. The model controlled for time periods to account for variants (Omicron emergence) and COVID-19 epidemic curves and county to account for county variations in case management and contact elicitation practices.

We estimated the adjusted odds ratios (aOR) and confidence intervals (CI) using multivariate logistic regression (p<0.05 was defined statistically significant and p<0.1 approaching significance are reported). Pearson’s *χ*^2^ test was utilized to compare differences between groups prior to multivariate regression. The goodness of statistics (*χ*^2^ test and Hosmer Lemeshaw statistic) were utilized to ensure the validity of the final multivariate regression model. SAS version 9.4 was utilized for all analyses. The New York State Department of Health institutional review board determined this surveillance activity was exempt from review and the need for patient consent.

## Results

**Fig 1:**
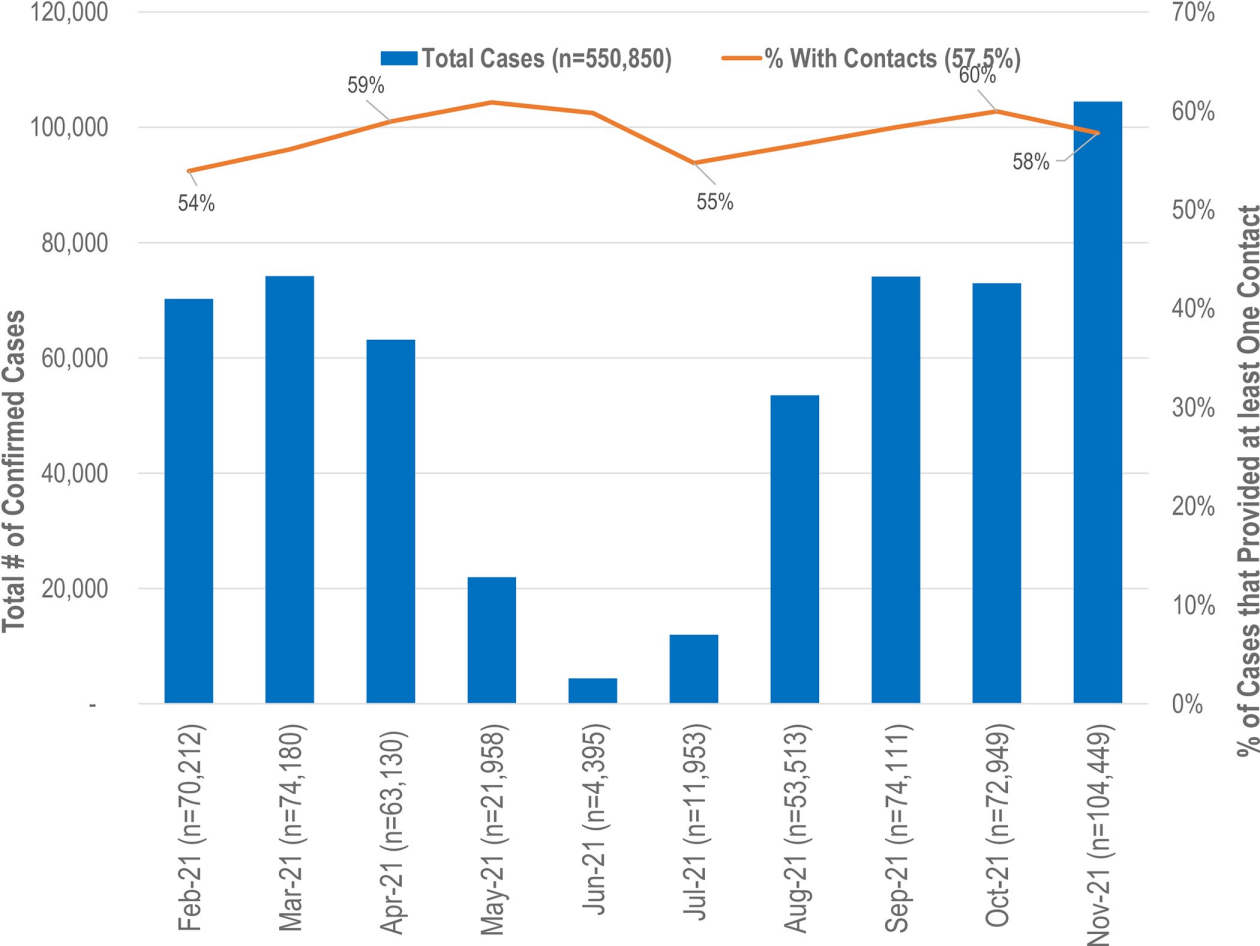
Confirmed cases and the percent with at least one contact: February 1, 2021 to November 30, 2021.

There were 550,850 confirmed cases that completed case interviews during the period, ranging from only 4,395 in June 2021 (seasonality) to 104,449 in November 2021 as Omicron surged (Fig 1). Of these, 316,645 (57.5%) provided at least one contact and varied between a low of 54% in February 2021 to a high of 60% in October 2021.

Table 1 displays descriptive statistics for those with and without contacts and there were significant differences (*χ*^2^ test). While only 48.7% of those aged 65 and older provided at least one contact, for the school aged population it was over 60% (63.3% for 5 to 11 and 65.7 for 12 to 17 years old children). Despite small in numbers, Native Americans and Hawaiian/Pacific Islanders had the highest percentages providing contacts at over 60 percent while Asians had the lowest at 53.4%. For gender, persons identifying as other or transgender, although a small population representing less than 1%, had the highest percentage (62.7%) providing contacts and males the lowest (55.6%). Those boosted were the most likely to provide contacts at almost 60% and unvaccinated the least likely at 57%—although not a large difference in percentage terms. A very high proportion of pregnant persons, 66.4%, provided at least one contact. Only 54.8% of those not associated with a K-12 setting provided contacts while 66.7% of those associated with a K-12 setting (students, staff, or visitors) provided a contact. Persons from two to four person households represented 73% of those that provided contacts, while they represented only 61.2% of those that did not provide contacts. Consequently, 61.7% of those in two to four person households provided at least one contact. Most persons in the study population were in single family households (82.5% of those providing contacts and 79.5% of those not providing contacts) and persons in single family households had a higher percent providing contacts relative to those in multi-unit households (58.4% for single family compared to 53.5% for multi-unit households). The average median income of households providing contacts was lower at $78,000 compared to $82,000 for those providing contacts and was significantly different.

**Table 1:** Characteristics of cases with and without contacts: February 1, 2021-November 30, 2021.

	#Cases Without Contacts (%)	#Cases With Contacts (%)	% Provided Contacts	*χ*^2^ *P* Value
Total	234,205(100)	316,645(100)	57.5	
**Age Categories**				
0–4 Years	9,823(4.2)	10,746(3.4)	52.2	< .0001
5–11 Years	19,320(8.2)	33,358(10.5)	63.3	
12–17 Years	17,379(7.4)	33,310(10.5)	65.7	
18–49 Years	112,732(48.1)	157,302(49.7)	58.3	
50–64 Years	48,800(20.8)	57,152(18.0)	53.9	
65 Plus	26,151(11.2)	24,777(7.8)	48.7	
**Race/Ethnicity**				
White	167,259(71.4)	233,534(73.8)	58.3	< .0001
Asian	6,877(2.9)	7,887(2.5)	53.4	
Black	20,542(8.8)	24,936(7.9)	54.8	
Hawaiian/Pacific Islander	175(0.1)	285(0.1)	62.0	
Hispanic	31,410(13.4)	39,179(12.4)	55.5	
Native American	926(0.4)	1,412(0.4)	60.4	
Other^a^	7,016(3.0)	9,412(3.0)	57.3	
**Gender**				
Female	118,815(50.7)	171,802(54.3)	59.1	< .0001
Male	115,236(49.2)	144,594(45.7)	55.6	
Non-Binary	33(0.0)	46(0.0)	58.2	
Other^b^	121(0.1)	203(0.1)	62.7	
**Vaccine Status**				
Boosted	1,612(0.7)	2,404(0.8)	59.9	< .0001
Primary Series	46,462(19.8)	66,291(20.9)	58.8	
Partial	12,090(5.2)	16,789(5.3)	58.1	
Unvaccinated	174,041(74.3)	231,161(73.0)	57.0	
**Symptoms**				
Symptoms (yes)	232,282(99.2)	315,461(99.6)	57.6	< .0001
Number of Symptoms, **mean (SD)**	3.6(3.2)	4.2(3.4)		< .0001
**Pregnancy**	1,969(0.8)	3,895(1.2)	66.4	< .0001
**Underlying Conditions**	80,898(34.5)	122,281(38.6)	60.2	< .0001
**Repeat Cases**	2,359(1.0)	2,852(0.9)	54.7	< .0001
**Hospitalization within 7 Days**	6,865(2.9)	7,573(2.4)	52.5	< .0001
**Setting Type**				
Non-K-12 School Setting	193,215(82.5)	234,714(74.1)	54.8	< .0001
K-12 School Setting	40,990(17.5)	81,931(25.9)	66.7	
Student	31,920(13.6)	63,101(19.9)	66.4	
Staff	3,798(1.6)	7,971(2.5)	67.7	
Visitor	311(0.1)	1,057(0.3)	77.3	
**Household Residents**				
One	42,972(18.3)	15,241(4.8)	26.2	< .0001
2–4	143,240(61.2)	231,065(73.0)	61.7	
5–6	38,831(16.6)	59,303(18.7)	60.4	
7–10	8,559(3.7)	10,493(3.3)	55.1	
11 >	603(0.3)	543(0.2)	47.4	
**Housing Type**				
Multi-family Housing	48,068(20.5)	55,338(17.5)	53.5	< .0001
Single-family Housing	186,137(79.5)	261,307(82.5)	58.4	
**Median Household Income per 10,000**				
Median Income, **mean (SD**)	8.2(3.4)	7.8(3.3)		< .0001
**Period**				
2/1/21-6/25/21	101,033(43.1)	132,336(41.8)	56.7	< .0001
6/26/21-8/15/21	14,734(6.3)	18,934(6.0)	56.2	
8/16/21-9/15/21	29,728(12.7)	38,705(12.2)	56.6	
9/16/21-10/15/21	30,867(13.2)	45,517(14.4)	59.6	
11/16/21-11/30/21	57,843(24.7)	81,153(25.6)	58.4	

^a^ Other was an option in the drop-down selection list for race.

^b^ These are persons that reported gender as other, transgender (male or female).

**Fig 2:**
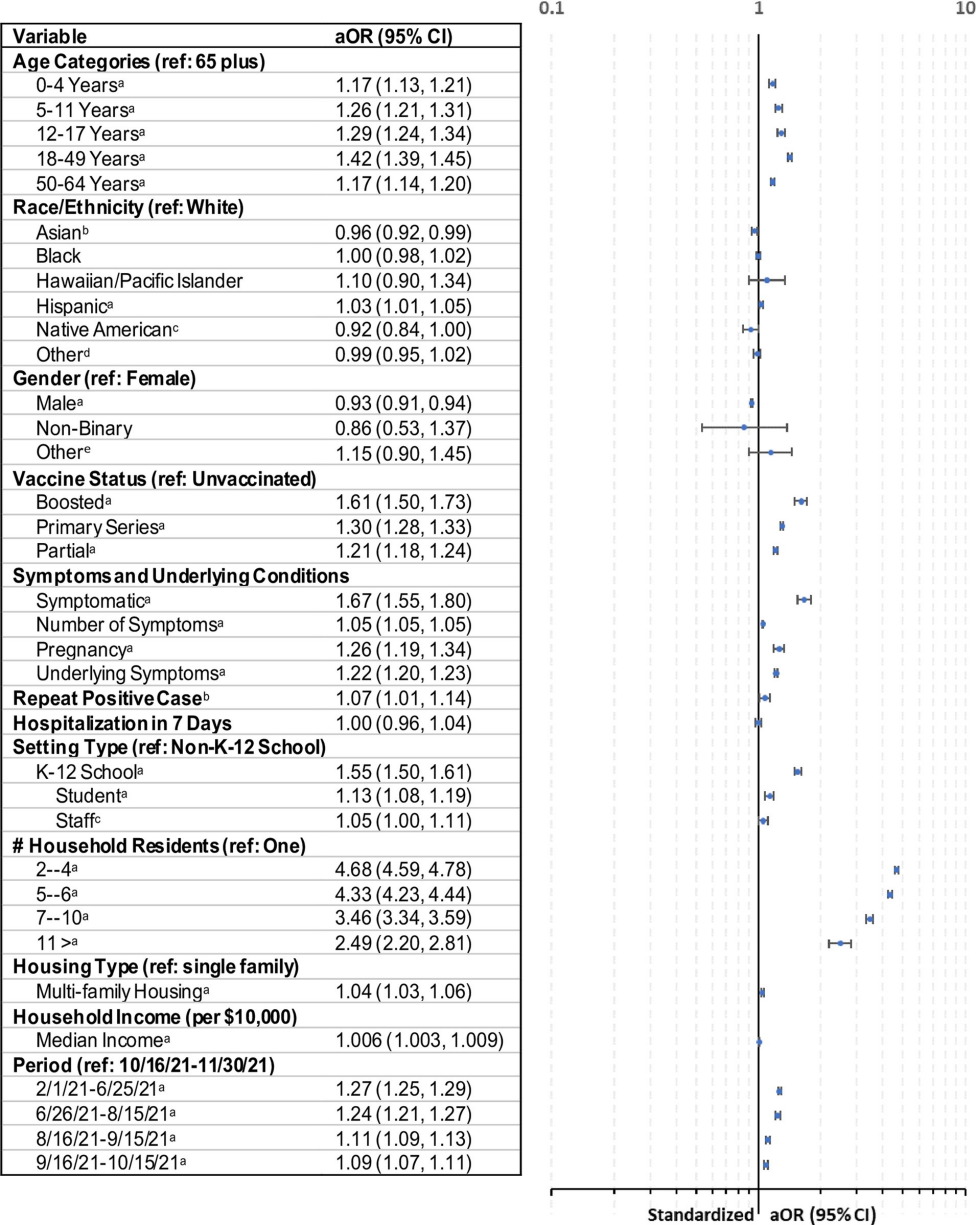
Cases providing at least one contact: Multivariate logistic regression of adjusted odds ratios (aOR) and confidence intervals (CI): February 1, 2021-November 30, 2021. Likelihood Ratio p-value = <0.0001; Hosmer and Lemeshow Goodness-of-Fit: p-value = 0.1414. The model included a county variable (coefficients not presented) to account for county variation in case management practices. ^a^p<0.01; ^b^p<0.05; ^c^p<0.10; ^d^Other was an option in the drop-down selection list for race; ^e^These are persons that reported gender as other, transgender (male or female).

Fig 2 provides aORs and CIs for various sub-groups examined. School-age persons (aOR, 1.17–1.39: 95% CI, 1.21–1.34) and adults 18 to 49 years (aOR, 1.42; 95% CI, 1.39–1.45) were the most likely to provide contacts relative to those 65 and older. School aged persons with aOR 1.26 (95% CI, 1.21, 1.31) for 5 to 11 years and 1.29 for 12 to 17 years (95% CI, 1.24–1.34) were also more likely to provide contacts. Compared to White persons, Asian persons (aOR, 0.96; 95% CI, 0.96–0.99) and Native Americans (aOR, 0.92; 95% CI, 0.84–1.00) were less likely to be while Hispanic persons (aOR, 1.03; 95% CI, 1.01–1.05) were more likely to provide contacts. Persons reported as female were more likely to provide contacts than persons reported as male (aOR, 0.93; 95% CI, 0.94–0.91). In contrast, there was no significant difference in contacts provision for persons that reported non-Binary or other as gender relative to persons that reported as male.

Compared to unvaccinated cases, contact elicitation was most successful with boosted individuals (aOR, 1.61; 95% CI, 1.50–1.73), followed by primary series (aOR 1.3; 95% CI, 1.28–1.33), and partially vaccinated (aOR, 1.21; 95% CI, 1.18–1.24). Repeat cases (aOR, 1.07; 95% CI, 1.01–1.14) were more likely to provide contacts compared to persons with only one COVID diagnosis. Those presenting symptoms (aOR, 1.67; 95% CI, 1.55–180) were more likely to report contacts than asymptomatic cases. Each additional symptom reported was associated with a 5 percent (95% CI, 4.9%-5.3%) increase in providing contacts. Pregnancy (aOR, 1.26; 95% CI, 1,19–1.34) and underlying conditions (aOR, 1.22; 95% CI, 1.20–1.23) increased the likelihood of providing contacts. Hospitalization, at an odds ratio of almost one, was not statistically significant. This is, in contrast to bivariate values in Table 1 where 2.9% of those that did not provide contacts were hospitalized compared to only 2.4% of those that provided contacts.

Those in K-12 school settings (aOR, 1.55; 95% CI, 1.50–1.61) and students (aOR, 1.13; 95% CI, 1.08–1.19) were more likely to provide contacts than those in non-K-12 school settings. For household size, aOR ranged from 2.49 to 4.7 (95% CI, range 2.20 to 4.78) for households with two or more persons relative to one-person households. The aOR was highest for the 2 to 4 person households and it declined as the household size increased. Household type aOR was 1.04 (95% CI, 1.01–1.06) for multi-family housing compared to single-family homes. Each additional $1,000 increase in residential zip code median income increased the likelihood of providing a contact by 0.6% percent (95% CI, 0.3%-0.9%) with an aOR 1.006 (95% CI, 1.003, 1.009).

S3 Fig in S1 File provides sensitivity analysis with aORs and CIs for various sub-groups examined prior to excluding persons with missing critical variables (age, gender, race/ethnicity, and household size). Results and conclusions remain consistent with that in Fig 2.

## Discussion

In NYS during the period observed, only 57.5% of confirmed COVID-19 cases with completed interviews, provided at least one contact. School-aged individuals, adults aged 18–49 years, boosted, symptomatic, pregnant, with underlying conditions, those living in 2–4 person households, and those in single family households were most likely to provide contacts, while individuals who were Asian, male, and unvaccinated were less likely. Therefore, while contacts were not elicited from all cases, there was success in eliciting contacts from prioritized groups among individuals interviewed. The proportion of interviewed cases that provided contacts in NYS was comparable to that in NYC and fell within the range observed elsewhere [5, 8–11]. Understanding the population groups that provided contacts and those who may require targeted outreach for contact-tracing is essential at reducing the percentage of interviewed cases who report zero contacts, which was documented to be over 40% in this study. Furthermore, examining the associations controlled for other factors is important as is observed for the household size, hospitalizations, and zip code level income.

The subgroups more likely to provide contacts included those prioritized [7, 13] for contact tracing, indicating success in reaching them. Adults aged 18–49, school-age individuals, and those in K-12 school settings were persons “working or visiting educational institutions.” “Members of a large household living in close living quarters” were captured using household size and housing type (close living quarters using those in multi-family housing) and were more likely to provide contacts once controlled for household size. The bivariate percent of those that provided contacts was higher at 58.4% for those in single family housing compared to 53.5% for those in multi-family housing (Table 1). However, the aOR was 1.04 for multi-family housing (relative to single family housing) indicating that once controlled for other variables, there was success in eliciting contacts from those living in close quarters (Fig 2).

There was success in eliciting contacts from prioritized pregnant and high-risk individuals (with underlying conditions). Boosted individuals may include high-risk persons due to their early eligibility. Similarly, while not prioritized per se repeated cases may be at higher risk or alternately prior experiences may impact their willingness to provide contacts. Some non-prioritized groups such as boosted, fully vaccinated, Hispanics and persons from higher-income zip codes were also more likely to provide contacts while hospitalization (a prioritized group) was not significant. Absence of differentials for hospitalized persons may be due to association of hospitalization with other factors such as pre-existing conditions. Alternately, could be real with no difference for hospitalized, given bivariate statistics indicated a slightly higher share of those not providing contacts were hospitalized (2.9% of those not providing contacts compared to 2.4% of those providing contacts). This could be due to challenges in reaching hospitalized persons.

High level of contact elicitation from those associated with K-12 settings is not surprising given school contact elicitation requirements at the time [19] and indicates success in implementing those requirements. The shift in the association with income when it is examined in isolation (median zip code level income was higher for those not providing contacts in Table 1) versus when controlled for other variables (those from higher income zip codes were more likely to provide contacts in Fig 2) indicates that adjusting for other factors is important. It also indicates that socio-economic factors as measured by income may play a role.

This study has several limitations. First, cases derived from home testing were not included; however, this period is prior to widespread home-test usage [16]. Second, the study was limited to cases with completed interviews in home-based residential settings and with non-missing values for demographic elements. The groups excluded from analysis had substantially lower contact elicitation. Only 4.3% of those not interviewed and 16% of those in excluded residential settings provided at least one contact (S1 Table in S1 File). Cases with missing values (5.6%) that were excluded from the analysis had no impact as a multivariate model including them resulted in the same conclusions (S3 Fig in S1 File). Finally, the analysis period was when vaccination was available, and the contact tracing program was fully functional. Therefore, the characteristics of those providing contacts in other periods may be different.

There are several areas that warrant further investigation. First is, why some non-prioritized groups provided contacts while those hospitalized, a priority group, did not need exploration to identify potential reasons. Second, there is a need to understand the case elicitation patterns and characteristics associated with groups excluded from this analysis such as those without complete interviews and those in congregate settings. For those not interviewed, a question would be investigating why they were not interviewed given the role interviews play in contact elicitation. Finally, the association with income needs further examination particularly given the shift observed between bivariate and multivariate analysis.

## Conclusion

In conclusion, while contacts were not elicited from all cases, there was success in eliciting contacts from prioritized groups among individuals interviewed. Targeted approaches for those less likely to provide contacts such as for hospitalized, Asian, male, and unvaccinated populations should be considered in future contact elicitation efforts.

## Supporting information

S1 File Supplemental materials for the manuscript entitled contact tracing: Characteristics of COVID-19 cases that provided contacts. (PDF)
