# Supplementary material for: Contact tracing: Characteristics of COVID-19 cases that provided contacts
Source: PLoS One. 2023 Nov 2;18(11):e0293208. doi: 10.1371/journal.pone.0293208 (PMC10621982; doi:10.1371/journal.pone.0293208)
Supplement: S1 File — (PDF) [file pone.0293208.s001.pdf]

Supplemental Materials for the manuscript entitled Contact Tracing: Characteristics of COVID-19 cases that provided contacts

Vajeera Dorabawila; Doris Maduka; Virgile Barnes, Nirmala Ramesh; Dina Hoefer

Supplemental Methods: Dataset construction

In New York State (NYS), applicable information on positive COVID-19 laboratory tests in Electronic Clinical Laboratory System (ECLRS) were automatically transmitted to the Communicable Disease Case Management System (CDCMS) every 30 minutes. In CDCMS, if the positive test specimen collection date was greater than 90 days from the specimen collection date of the first positive result or the most recent case specimen collection date, a new case was created[1]. Additionally, Local Health Departments (LHDs) could add cases based on positive tests not captured in ECLRS. LHD case interviewers conducted case interviews primarily via phone interviewers and other household members including sexual partners may have been present during the interviews. A contact is defined as persons within 6 feet for 15 cumulative minutes during a 24-hour period of the case while the person was potentially infectious starting from 2 days before illness onset and for asymptomatic persons 2 days prior to specimen collection date[2]. The NYS Immunization Information System (NYSIIS) contains COVID-19 vaccination data for NYS (others than New York City) residents. The Health Electronic Response Data System (HERDS) is a statewide, daily electronic survey of hospitals of COVID-19 hospitalizations at inpatient facilities.

The vaccine information system (NYSIIS) and laboratory data (ECLRS) were matched to case data (CDCMS) using a deterministic algorithm based on first name, last name, and date of birth. This was then matched to hospitalizations (HERDS) using first and last initial, date of birth, gender, and zip code. American Community Survey 5-year estimates was matched using residential zip code to obtain zip code median income.

This analysis is restricted to the period from February 1, 2021 to November 2021. In NYS, February 2021 was the first full month when vaccines were available[3]. As vaccination is a variable of interest, the analysis period start was February 1,2021. The contact tracing program in NYS was initiated in May 2020, and the program was ramped up in the Summer 2020. In January 2022, following CDC guidelines on contact tracing, NYS gave LHDs the ability to suspend their contact tracing program[4]. However, by December 2021 due to the surge in COVID-19, interview completion rates and contact elicitation declined substantially (S1 Fig). Therefore, the end of the period was restricted to when case completion rates were relatively high to prevent bias. This resulted in 777,225 total confirmed cases for analysis (S2 Fig). This period is prior to when home test usage became prevalent[5].

Cases were restricted to those with complete interviews, valid zip codes, living in single family or multi-family residential settings and with non-missing values for age, race/ethnicity, gender, and household size (S2 Fig). Cases without complete interviews were excluded given interviews were an integral component of contact elicitation. As residential zip code was utilized to match with American Community Survey (ACS), those missing zip code would miss median zip code and were excluded. Cases from congregate and other unspecified housing were excluded given the contact tracing processes may be different for those persons. Of the 777,225 confirmed cases, 12.5% had incomplete interviews, 10.6% were in congregate housing, 0.6% had invalid and inconsistent zip codes and 5.6% had missing age, race, ethnicity, or gender. This resulted in 550,854 cases for analysis, of which 57.5 percent provided at least one contact. S1 Table compares the characteristics of the persons included in the main analysis with those excluded (not interviewed and those from congregate or unspecified residential settings).

A multivariate logistic regression model was also developed including cases with missing zip code, age, race, ethnicity, and gender (S3 Fig). The conclusions are consistent with figure 3 presented in the main paper. Furthermore, the intercept values for in the main model (figure 3 intercept -2.33) and supplemental model (S3 fig intercept -2.4) were very close with the same level of significance (p value<0.001). These results further supported the decision to exclude these persons from the main analysis.

S1 Fig: May 2020-August 2022: Number of Confirmed Cases, Percent Interview Completed and Percent that Provided at Least One Contact.

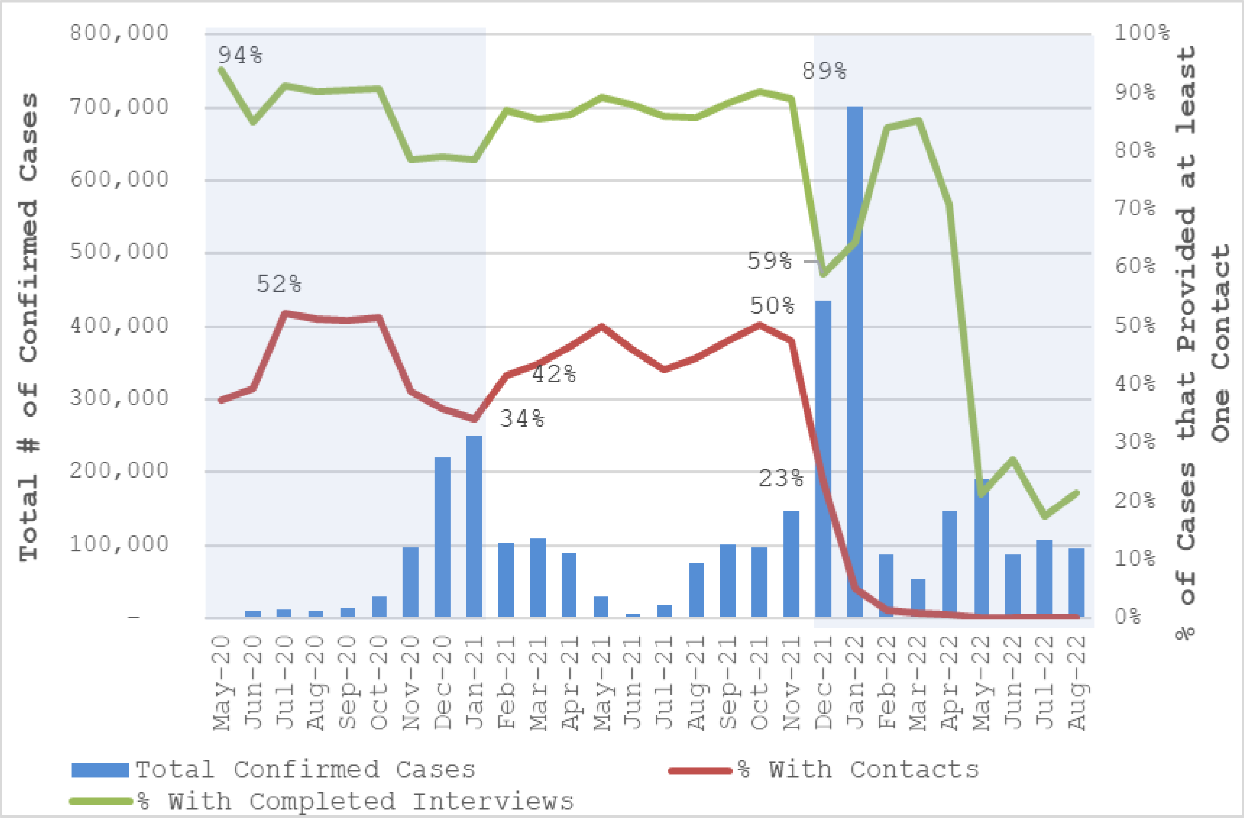

S2 Fig: COVID-19 Confirmed Cases in New York State from May 1 to August 30, 2022 and February 1, 2021 to November 30, 2021

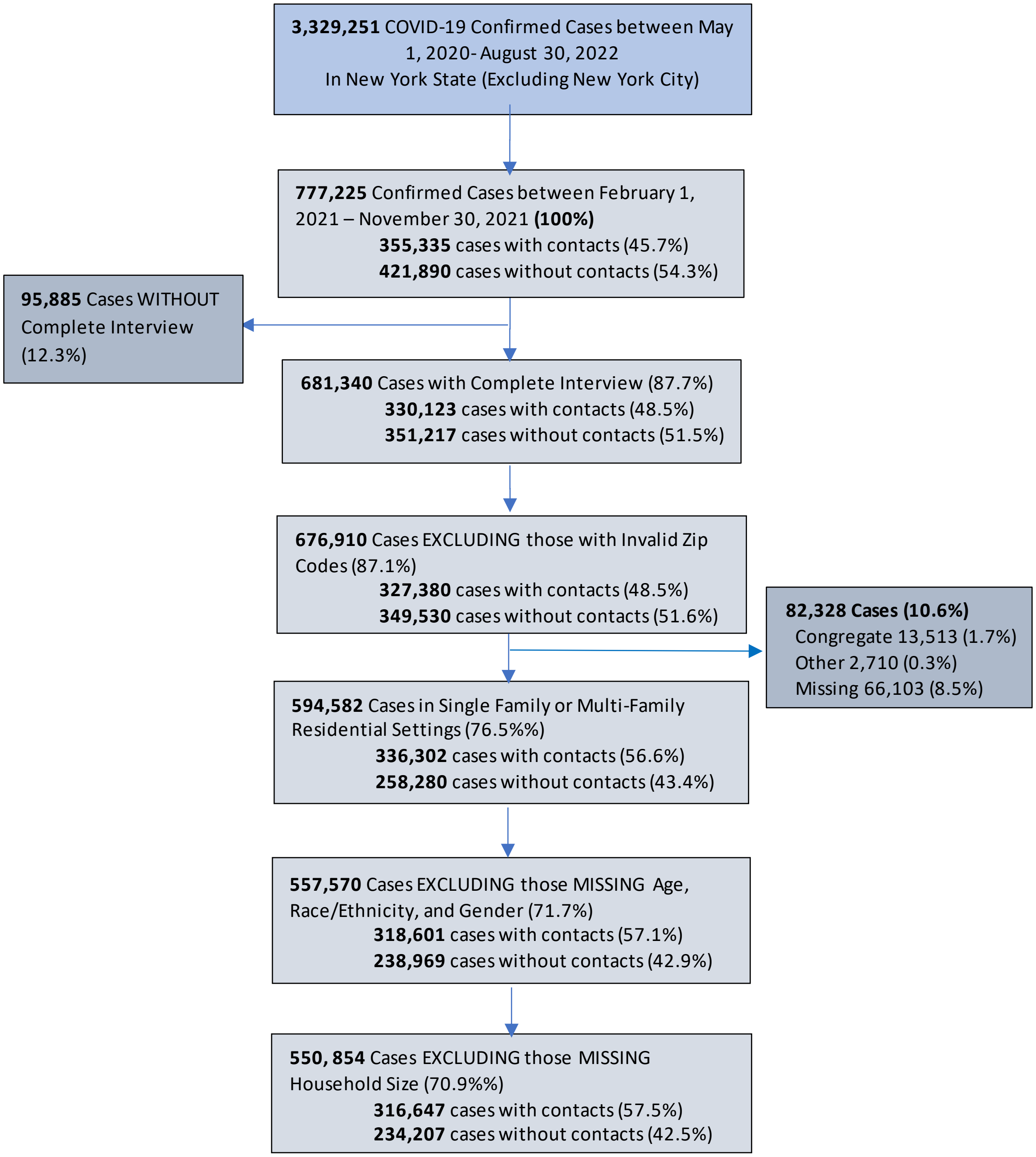

S1 Table: Comparison of Characteristics of Cases Interviewed in the Analysis Sample, Cases not Interviewed and Cases Interviewed from Other, Congregate and Missing Residential Settings.

	Analysis Population			Groups Excluded from the Analysis					
	Cases Interviewed			Cases Not Interviewed			Cases Interviewed (Congregate, Other and Missing Residential Settings)		
	#Cases Without Contacts (%)	#Cases With Contacts (%)	% Provided Contacts	#Cases Without Contacts (%)	#Cases With Contacts (%)	% Provided Contacts	#Cases Without Contacts (%)	#Cases With Contacts (%)	% Provided Contacts
**Total**	**234,207**	**316,647**	**57.5**	**91,767**	**4,118**	**4.3**	**70,360**	**13,441**	**16.0**
**Age Categories**									
0-4 Years	9,823(4.2)	10,746(3.4)	52.2	2,458(2.7)	106(2.6)	4.1	1,954(2.8)	411(3.1)	17.4
5-11 Years	19,320(8.2)	33,358(10.5)	63.3	4,051(4.4)	697(16.9)	14.7	3,749(5.3)	1,524(11.3)	28.9
12-17 Years	17,379(7.4)	33,310(10.5)	65.7	5,719(6.2)	750(18.2)	11.6	4,112(5.8)	1,413(10.5)	25.6
18-49 Years	112,733(48.1)	157,303(49.7)	58.3	48,519(52.9)	1,583(38.4)	3.2	34,847(49.5)	7,309(54.4)	17.3
50-64 Years	48,801(20.8)	57,153(18.0)	53.9	17,615(19.2)	575(14.0)	3.2	12,581(17.9)	1,649(12.3)	11.6
65plus	26,151(11.2)	24,777(7.8)	48.7	12,648(13.8)	367(8.9)	2.8	12,734(18.1)	1,052(7.8)	7.6
Missing	0(0.0)	0(0.0)	0.0	757(0.8)	40(1.0)	5.0	383(0.5)	83(0.6)	17.8
**Race/Ethnicity**									
Hispanic	31,410(13.4)	39,180(12.4)	55.5	6,612(7.2)	292(7.1)	4.2	5,458(7.8)	1,762(13.1)	24.4
Asian	6,877(2.9)	7,887(2.5)	53.4	1,747(1.9)	77(1.9)	4.2	1,257(1.8)	407(3.0)	24.5
Black	20,542(8.8)	24,936(7.9)	54.8	4,197(4.6)	175(4.2)	4.0	4,029(5.7)	1,104(8.2)	21.5
White	167,261(71.4)	233,535(73.8)	58.3	25,742(28.1)	1,459(35.4)	5.4	26,462(37.6)	7,690(57.2)	22.5
Native	926(0.4)	1,412(0.4)	60.4	146(0.2)	9(0.2)	5.8	181(0.3)	63(0.5)	25.8
Hawaiian	175(0.1)	285(0.1)	62.0	59(0.1)	3(0.1)	4.8	46(0.1)	18(0.1)	28.1
Other	7,016(3.0)	9,412(3.0)	57.3	3,564(3.9)	136(3.3)	3.7	2,827(4.0)	390(2.9)	12.1
Missing	0(0.0)	0(0.0)	0.0	49,700(54.2)	1,967(47.8)	3.8	30,100(42.8)	2,007(14.9)	6.3
**Gender**									
Female	118,817(50.7)	171,803(54.3)	59.1	43,377(47.3)	2,297(55.8)	5.0	33,409(47.5)	6,967(51.8)	17.3
Male	115,236(49.2)	144,595(45.7)	55.6	46,162(50.3)	1,715(41.6)	3.6	35,830(50.9)	6,358(47.3)	15.1
Non-Binary	33(0.0)	46(0.0)	58.2	2(0.0)	0(0.0)	0.0	4(0.0)	4(0.0)	50.0
Other	121(0.1)	203(0.1)	62.7	520(0.6)	8(0.2)	1.5	370(0.5)	24(0.2)	6.1

	Analysis Population			Groups Excluded from the Analysis					
	Cases Interviewed			Cases Not Interviewed			Cases Interviewed (Congregate, Other and Missing Residential Settings)		
	#Cases Without Contacts (%)	#Cases With Contacts (%)	% Provided Contacts	#Cases Without Contacts (%)	#Cases With Contacts (%)	% Provided Contacts	#Cases Without Contacts (%)	#Cases With Contacts (%)	% Provided Contacts
**Total**	**234,207**	**316,647**	**57.5**	**91,767**	**4,118**	**4.3**	**70,360**	**13,441**	**16.0**
Missing	0(0.0)	0(0.0)	0.0	1,706(1.9)	98(2.4)	5.4	747(1.1)	88(0.7)	10.5
**Vaccine Status**									
Boosted	1,612(0.7)	2,404(0.8)	59.9	543(0.6)	17(0.4)	3.0	648(0.9)	72(0.5)	10.0
Primary Series	46,463(19.8)	66,292(20.9)	58.8	13,890(15.1)	592(14.4)	4.1	12,347(17.5)	2,254(16.8)	15.4
Partial	12,090(5.2)	16,789(5.3)	58.1	4,386(4.8)	181(4.4)	4.0	4,066(5.8)	777(5.8)	16.0
Unvaccinated	174,042(74.3)	231,162(73.0)	57.0	72,948(79.5)	3,328(80.8)	4.4	53,299(75.8)	10,338(76.9)	16.2
**Symptoms**									
Symptomatic	232,284(99.2)	315,462(99.6)	57.6	3,746(4.1)	766(18.6)	17.0	20,774(29.5)	11,776(87.6)	36.2
Number of Symptoms	3.6(3.2)	4.2(3.4)		0.1(0.8)	0.7(2.0)		0.8(1.9)	3.0(3.0)	
**Pregnancy**	1,969(0.8)	3,895(1.2)	66.4	175(0.2)	14(0.3)	7.4	166(0.2)	84(0.6)	33.6
**Underlying Symptoms**	80,900(34.5)	122,282(38.6)	60.2	1,397(1.5)	294(7.1)	17.4	5,746(8.2)	3,208(23.9)	35.8
**Repeat Positive Case**	2,359(1.0)	2,852(0.9)	54.7	1518(1.7)	57(1.4)	3.6	1,197(1.7)	151(1.1)	11.2
**Hospitalization Within 7 Days**	6,865(2.9)	7,573(2.4)	52.5	8,060(8.8)	359(8.7)	4.3	6,328(9.0)	679(5.1)	9.7
**Setting Type**									
Non-School	193,217(82.5)	234,716(74.1)	54.8	90,985(99.1)	3,662(88.9)	3.9	68,159(96.9)	10,881(81.0)	13.8
School	40,990(17.5)	81,931(25.9)	66.7	782(0.9)	456(11.1)	36.8	2,201(3.1)	2,560(19.0)	53.8
**Household Residents**									
1	42,972(18.3)	15,241(4.8)	26.2	361(0.4)	53(1.3)	12.8	3,530(5.0)	1,812(13.5)	33.9
2--4	143,242(61.2)	231,067(73.0)	61.7	2,082(2.3)	547(13.3)	20.8	4,787(6.8)	6,099(45.4)	56.0
5--6	38,831(16.6)	59,303(18.7)	60.4	654(0.7)	138(3.4)	17.4	1,134(1.6)	1,440(10.7)	55.9
7--10	8,559(3.7)	10,493(3.3)	55.1	170(0.2)	30(0.7)	15.0	496(0.7)	358(2.7)	41.9
11 >	603(0.3)	543(0.2)	47.4	23(0.0)	5(0.1)	17.9	386(0.5)	149(1.1)	27.9
Missing	0(0.0)	0(0.0)	0.0	88,477(96.4)	3,345(81.2)	3.6	60,027(85.3)	3,583(26.7)	5.6

	Analysis Population			Groups Excluded from the Analysis					
	Cases Interviewed			Cases Not Interviewed			Cases Interviewed (Congregate, Other and Missing Residential Settings)		
	#Cases Without Contacts (%)	#Cases With Contacts (%)	% Provided Contacts	#Cases Without Contacts (%)	#Cases With Contacts (%)	% Provided Contacts	#Cases Without Contacts (%)	#Cases With Contacts (%)	% Provided Contacts
**Total**	**234,207**	**316,647**	**57.5**	**91,767**	**4,118**	**4.3**	**70,360**	**13,441**	**16.0**
**Housing Type**									
Multi-family Housing	48,068(20.5)	55,338(17.5)	53.5	818(0.9)	131(3.2)	13.8	0(0.0)	0(0.0)	0.0
Single-family Housing	186,139(79.5)	261,309(82.5)	58.4	2,674(2.9)	578(14.0)	17.8	0(0.0)	0(0.0)	0.0
Congregate Housing	0(0.0)	0(0.0)	0.0	1,216(1.3)	79(1.9)	6.1	9,980(14.2)	3,934(29.3)	28.3
Other Housing	0(0.0)	0(0.0)	0.0	34(0.0)	8(0.2)	19.0	1,327(1.9)	1,414(10.5)	51.6
Missing	0(0.0)	0(0.0)	0.0	87,025(94.8)	3,322(80.7)	3.7	59,053(83.9)	8,093(60.2)	12.1
**Median Household Income per 10,000**									
Median Income, **mean (SD)**	8.2(3.4)	7.8(3.3)		8.8(3.8)	8.4(3.7)		7.8(3.5)	7.1(4.2)	
**Period**									
2/1/21-6/25/21	101,033(43.1)	132,336(41.8)	56.7	43,548(47.5)	1,843(44.8)	4.1	35,530(50.5)	6,686(49.7)	15.8
6/26/21-8/15/21	14,734(6.3)	18,934(6.0)	56.2	6,608(7.2)	174(4.2)	2.6	3,782(5.4)	622(4.6)	14.1
8/16/21-9/15/21	29,729(12.7)	38,705(12.2)	56.6	11,888(13.0)	404(9.8)	3.3	7,167(10.2)	1,202(8.9)	14.4
9/16/21-10/15/21	30,868(13.2)	45,518(14.4)	59.6	10,584(11.5)	657(16.0)	5.8	7,836(11.1)	1,365(10.2)	14.8
10/16/21-11/30/21	57,843(24.7)	81,154(25.6)	58.4	19,139(20.9)	1,040(25.3)	5.2	16,045(22.8)	3,566(26.5)	18.2

S3 Fig: Cases Providing at Least One Contact: Multivariate Logistic Regression Adjusted Odds Ratios (aOR) and Confidence Intervals (CI): February 1, 2021-November 30, 2021. Likelihood Ratio p-value= <0.0001; Hosmer and Lemeshow Goodness-of-Fit: p-value=0.1414. The model included a county variable (coefficients not presented) to account for county variation in case management practices. [a] p<0.01; [b] p<0.05; [c] p<0.10; [d] Other was an option in the drop-down selection list for race; [e] These are persons that reported gender as other, transgender (male or female).

Variable	aOR (96% CI)
**Age Categories (ref: 65 plus)**	
0-4 Years[a]	1.19 (1.15, 1.24)
5-11 Years[a]	1.28 (1.23, 1.32)
12-17 Years[a]	1.31 (1.26, 1.36)
18-49 Years[a]	1.43 (1.40, 1.46)
50-64 Years[a]	1.17 (1.14, 1.20)
Missing[a]	0.72 (0.64, 0.80)
**Race/Ethnicity (ref: White)**	
Asian[b]	0.97 (0.93, 1.00)
Black	1.01 (0.99, 1.03)
Hawaiian	1.10 (0.91, 1.34)
Hispanic[a]	1.04 (1.02, 1.06)
Missing[a]	0.82 (0.80, 0.84)
Native[c]	0.93 (0.85, 1.01)
Other[d]	0.99 (0.95, 1.02)
**Gender (ref: Female)**	
Male[a]	0.92 (0.91, 0.93)
Non-Binary	0.92 (0.59, 1.45)
Other[d]	1.06 (0.85, 1.32)
Missing[a]	0.72 (0.62, 0.84)
**Vaccine Status (ref: Unvaccinated)**	
Boosted[a]	1.60 (1.49, 1.71)
Primary Series[a]	1.30 (1.28, 1.32)
Partial[a]	1.21 (1.18, 1.24)
**Symptoms and Underlying Conditions**	
Symptomatic[a]	1.73 (1.62, 1.85)
Number of Symptoms[a]	1.05 (1.05, 1.05)
Pregnancy[a]	1.27 (1.20, 1.34)
Underlying Symptoms[a]	1.22 (1.21, 1.24)
Repeat Positive Case[c]	1.05 (1.00, 1.12)
Hospitalization Within 7 Days	1.00 (0.97, 1.04)
**Setting Type (ref: Non School)**	
School[a]	1.57 (1.51, 1.62)
Student[a]	1.13 (1.08, 1.18)
Staff[b]	1.06 (1.00, 1.11)
**# Household Residents (ref: One)**	
2--4[a]	4.69 (4.60, 4.78)
5--6[a]	4.32 (4.22, 4.42)
7--10[a]	3.46 (3.35, 3.59)
11 >[a]	2.41 (2.15, 2.71)
Missing[a]	1.41 (1.33, 1.48)
**Housing Type (ref: single family)**	
Multi-family Housing[a]	1.05 (1.03, 1.06)
**Household Income**	
Median Income (per $10,000) [a]	1.01 (1.01, 1.01)
**Period (ref: 10/16/21-11/30/21)**	
2/1/21-6/25/21[a]	1.26 (1.24, 1.28)
6/26/21-8/15/21[a]	1.22 (1.19, 1.25)
8/16/21-9/15/21[a]	1.10 (1.08, 1.12)
9/16/21-10/15/21[a]	1.08 (1.06, 1.10)

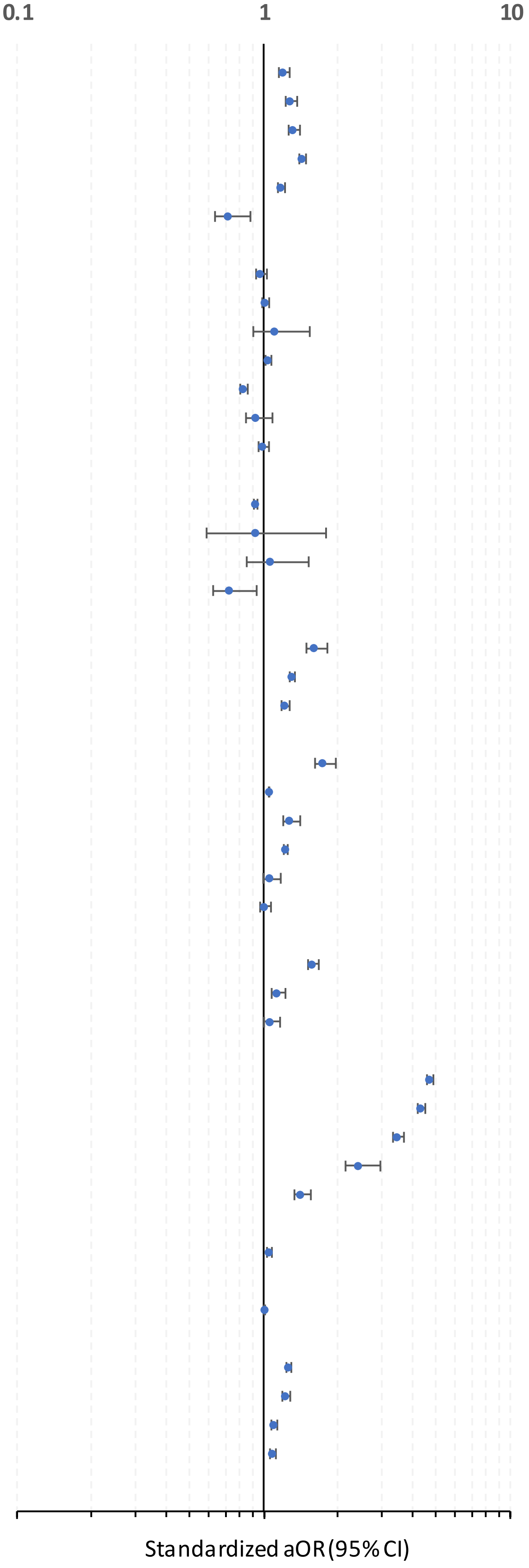

Supplemental References

1. Centers for Disease Control and Prevention. Coronavirus Disease 2019 (COVID-19) 2021 Case Definition. Accessed February 1, 2023. https://ndc.services.cdc.gov/case-definitions/coronavirus-disease-2019-2021/
2. Centers for Disease Control and Prevention. Interim Guidance on Developing a COVID-19 Case Investigation & Contact Tracing Plan. Accessed February 1, 2023. https://stacks.cdc.gov/view/cdc/88623
3. New York State Department of Health. Governor Cuomo Announces New Yorkers 30 Years of Age and Older Will Be Eligible to Receive COVID-19 Vaccine. Accessed February 1, 2023. https://www.governor.ny.gov/news/governor-cuomo-announces-new-yorkers-30-years-age-and-older-will-be-eligible-receive-covid-19
4. New York State Department of Health. Isolation: What to Do If You Test Positive, Were Exposed to Someone Who Tested Positive or Display COVID-19 Symptoms. Accessed February 1, 2023. https://coronavirus.health.ny.gov/isolation
5. Dorabawila V, Barnes V, Ramesh N et al. (2023) Comparison of COVID-19 home-testing and laboratory-based testing methods in New York State (excluding New York City) from November 2021 to April 2022. Front. Public Health. 2023; 11:1058644. doi:10.3389/fpubh.2023.1058644
